# OAF Blocks SIAH1‐Mediated Degradation of SCPX, a Therapeutic Strategy for MASLD

**DOI:** 10.1002/advs.77451

**Published:** 2026-08-29

**Authors:** Zongxi Li, Jingyue Song, Kaili Ma, Shunan Tang, Shuqian Wang, Wenya Ji, Chen Lu, Ting Zhang, Huaping Zheng, Xiaowei Zheng, Shanshan Lai, Cheng Deng

**Affiliations:** ^1^ Jiangsu Key Laboratory For Biodiversity and Biotechnology College of Life Sciences Nanjing Normal University Nanjing People's Republic of China; ^2^ Department of High Altitude Medicine Center For High Altitude Medicine College of Life Sciences National Clinical Research Center for Geriatrics West China Hospital Sichuan University Chengdu Sichuan People's Republic of China; ^3^ Institute of High Altitude Medicine High Altitude Medicine Key Laboratory of Sichuan Province West China Hospital Sichuan University Chengdu Sichuan People's Republic of China; ^4^ Department of Molecular Medicine and Surgery Karolinska Institute Stockholm Sweden; ^5^ Center For High Altitude Medicine Xining Qinghai People's Republic of China

**Keywords:** lipid metabolism, MASLD, OAF, SCPX, SIAH1

## Abstract

Metabolic dysfunction‐associated steatotic liver disease (MASLD) poses an increasing threat to global public health. Currently approved drugs for MASLD, such as resmetirom and semaglutide, have demonstrated clinical efficacy. However, their use is associated with notable adverse effects, including serious gastrointestinal reactions, metabolic and nutritional disturbances, and renal or urinary system disorders. The present study established that the expression levels of a liver‐secreted protein, the “out at first homolog” (*Oaf*) gene product, are closely associated with metabolic disorders in humans and mice. In a high‐fat diet (HD) feeding mouse model, *Oaf*‐deficiency exacerbated hepatic lipid accumulation, whereas intraperitoneal injection of OAF protein ameliorated the development of MASLD. Mechanistically, OAF binds to SCPX and acts as a chaperone, preventing SCPX from degradation by SIAH1‐mediated ubiquitination. This stabilizes SCPX and increases its abundance, thereby enhancing cholesterol transport, lipid esterification, and lipid excretion, while inhibiting lipid biosynthesis. In mice, deficiency of either *Oaf* or *Scpx* reduced these effects. Collectively, our findings identify OAF as a novel therapeutic target for the treatment of MASLD and provide a rationale for further drug development targeting human fatty liver diseases.

## Graphical abstract

This study shows OAF directly binds to SCPX and inhibits its interaction with the E3 ligase SIAH1, thereby protecting SCPX from ubiquitin‐dependent degradation and stabilizing its levels. This OAF‐SCPX axis represents a novel pathway in lipid homeostasis, highlighting OAF as a promising therapeutic candidate for MASLD.

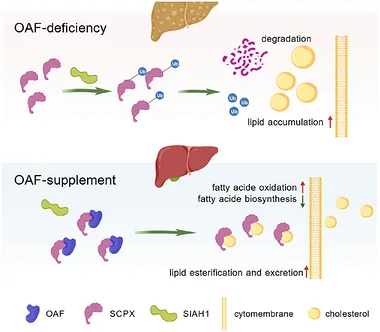

## 1 Introduction

Metabolic dysfunction‐associated steatotic liver disease (MASLD), previously known as non‐alcoholic fatty liver disease (NAFLD), is a chronic multisystem disorder characterized by hepatic lipid accumulation, inflammation, and, in some cases, progression to hepatocellular carcinoma [1]. Although the precise pathological mechanisms remain unclear, several contributing factors have been identified, including genetic predisposition, unhealthy dietary patterns, and physical inactivity [1, 2, 3]. MASLD often coexists with systemic metabolic abnormalities such as obesity and type 2 diabetes mellitus (T2DM), and its prevalence is rising globally, posing a major public health challenge [4]. Current pharmacological treatments for MASLD mainly comprise several therapeutic categories. Resmetirom, a thyroid hormone receptor‐β (THR‐β) agonist, and semaglutide, a glucagon‐like peptide‐1 receptor (GLP‐1R) agonist, have demonstrated clinical remission rates of 25.9%–29.9% at 52 weeks and 62.9% at 72 weeks, respectively, both of which are significantly higher than those achieved with placebo [5, 6]. Notably, resmetirom is the first approved agent directly targeting MASH with anti‐fibrotic effects, whereas semaglutide provides additional cardiovascular and renal benefits beyond glycemic control and weight reduction [7]. Furthermore, tirzepatide, a dual glucose‐dependent insulinotropic polypeptide (GIP) and GLP‐1 receptor agonist, has been approved for the treatment of type 2 diabetes and is considered a promising therapeutic candidate for MASLD [7]. In contrast, orlistat indirectly slows disease progression by inhibiting gastrointestinal lipase activity and reducing intestinal absorption of dietary fats [8]. Despite these important clinical advances, substantial unmet needs remain. A considerable proportion of patients fail to achieve disease remission, with approximately 37% of semaglutide‐treated patients and nearly 70% of resmetirom‐treated patients showing inadequate therapeutic responses. In addition, the efficacy of current therapies in improving liver fibrosis remains limited and variable, while the long‐term durability of treatment benefits has yet to be fully established. Furthermore, gastrointestinal adverse events may compromise treatment adherence and patient tolerability [5, 6, 9, 10]. Given the multifactorial and complex pathogenesis of MASLD, the identification of novel therapeutic targets and adjunctive treatment strategies remains an important research priority. Collectively, these limitations underscore the urgent need for the development of more effective and durable therapeutic interventions [11].

A gene named “out at first” (*Oaf*) was initially discovered in *Drosophila melanogaster*, where zygotic transcription appeared in small clusters of cells in the majority of segments during germ band extension and later in a pattern of segmental repeats in the central nervous system (CNS) during embryonic development. It is also expressed in the gonads of embryos, larvae, and adult flies of both sexes [12]. OAF protein is highly conserved across the animal kingdom (Figure S1). According to the Human Protein Atlas, OAF is abundantly expressed in the liver and gastrointestinal tract, and bioinformatic predictions suggest its secretory potential due to the presence of an N‐terminal signal peptide. In the liver, OAF is broadly expressed across multiple cell types, with particularly high expression levels observed in hepatocytes (https://www.proteinatlas.org/ENSG00000184232‐OAF, https://bis.zju.edu.cn/MCA/search2.html). Several studies have implicated OAF in diverse physiological and pathological processes, including corneal and lens tissue separation, pulmonary tuberculosis, cutaneous T‐cell lymphoma invasion and metastasis, amyloidogenesis, asthma, endocytosis of albumin, phthisis during COVID‐19, and drug‐induced liver injury [13, 14, 15, 16, 17, 18]. However, the underlying mechanisms remain poorly understood, and the biological functions of OAF are largely uncharacterized. Structurally, OAF has been proposed as a novel member of the BRICHOS domain‐containing chaperone protein family. This classification is based on key structural features, including: (1) an N‐terminal signal peptide for secretion; (2) a predicted furin‐like cleavage site flanked by two anti‐parallel β‐strands; and (3) a conserved disulfide bridge between α‐helix 2 and β‐strand 4, along with a highly conserved aspartic acid located at the end of β‐strand 2 [19]. While these features suggest a potential molecular chaperone function of OAF, this role remains to be experimentally validated.

Sterol carrier protein X (SCPX) is a 58 kDa precursor protein with thiolase and SCP2 domains. After import, it is processed in peroxisomes, generating separate 46 kDa thiolase and 13 kDa SCP2 proteins [20]. SCPX/SCP2 is implicated in modulating diverse lipid metabolic pathways relevant to MASLD, such as fatty acid, fatty acyl‐CoA, phospholipid, and cholesterol transfer between membranes and mitochondria [21, 22]. For instance, *Scpx/Scp2*‐deficient mice exhibited increased accumulation of cholesterol and glycerides in hepatocytes when fed a high‐cholesterol diet [23]. In primary hepatocytes, SCPX/SCP2 has been shown to participate in high density lipoprotein (HDL)‐mediated cholesterol uptake processes through liver fatty acid‐binding protein (FABP) [24, 25]. Moreover, a patient carrying a heterozygous mutation in *SCPX/SCP2* exhibited abnormal plasma levels of medium‐, long‐, and very long‐chain fatty acids, along with a reduced number of peroxisomes and altered mitochondrial fatty acid β‐oxidation [26, 27]. Recently, it has been reported that SCPX is subject to ubiquitin‐dependent degradation by the E3 ligase SIAH1, a process that exacerbates MASLD progression [28]. Therefore, SCPX/SCP2 is a crucial regulatory factor in the process of lipid metabolism and MASLD.

In our study, using *Oaf* knockout and protein administration models, as well as *Scpx* knockout and knockdown experiments, we demonstrate that OAF plays a critical role in hepatic lipid accumulation by directly binding to and stabilizing SCPX preventing its ubiquitin‐dependent degradation and thereby modulating the progression of MASLD. This work provides the first evidence that OAF acts as a potential molecular chaperone for SCPX, revealing a previously uncharacterized regulatory pathway in MASLD with therapeutic potential for metabolic diseases.

## 2 Results

### 2.1 Evolutionarily Conserved OAF Levels are Negatively Correlated with Serum Lipid Profiles in both Mice and Humans

**FIGURE 1:**
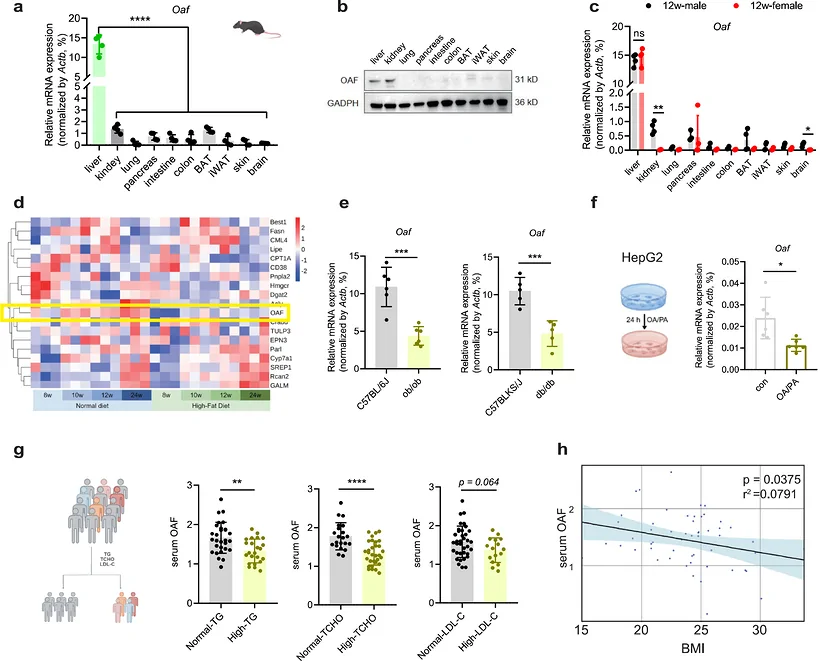
OAF expression is negatively correlated with liver steatosis. (a, b)qPCR and Western blot to measure *Oaf* mRNA expression (a) and protein levels (b) in various tissues of 8‐week‐old male C57BL/6J mice (N = 6 per group). (c) qPCR analysis of *Oaf* expression in different tissues of 12‐week‐old male and female C57BL/6J mice (N = 4 per group). (d) Analysis of GEO database (GDS6248) revealing *Oaf* expression in the livers of mice maintained on a HD or CD diet, data were analyzed using Bioladder (https://www.bioladder.cn). (e) qPCR analysis of *Oaf* mRNA expression in livers from 16‐week‐old male *ob/ob* and 18‐week‐old male *db/db* mice compared to normal controls (N = 5 per group). (f) qPCR analysis the *Oaf* mRNA expression after OA/PA supplementation in HepG2 cells. (g) ELISA analysis of serum OAF concentrations in clinical samples (high TG, TG > 5.6mmol/L, N = 29; Normal TG, N = 23; high TCHO, TCHO > 6.2mmol/L, N = 21; Normal TCHO, N = 31; high LDL‐C > 3.4mmol/L, N = 36; Normal LDL‐C, N = 16). (h) Correlation between serum OAF concentrations and body mass index (BMI) in human volunteers (N = 55). Data were obtained from independent samples and analyzed using GraphPad prism 9. Data are presented as mean ± SD. ^*^p < 0.05; ^**^ *p* < 0.01; ^***^ *p* < 0.001; ^****^ *p* < 0.0001, “ns” indicates notsignificant. Statistical analysis was performed using a two‐tailed unpaired Student's *t*‐test.

Comparative sequence analysis of OAF orthologs across representative animal phyla revealed strong evolutionary conservation, particularly within the BRICHOS domain, suggesting its involvement in fundamental biological functions (Figure S1a, b). Quantitative analysis confirmed that *Oaf* was highly transcribed in the livers of C57BL/6J mice compared to other tissues (Figure 1a, Figure S2a), and the corresponding protein was also abundantly expressed in the liver (Figure 1b, Figure S2b). Similarly. *Oaf* mRNA expression was high in liver of non‐mammalian vertebrates such as chickens (Figure S2c). Given the central role of the liver in metabolic regulation, we next investigated the relationship between OAF and metabolic dysfunction. Analysis of public transcriptomic data from the GEO database revealed that hepatic *Oaf* expression was lower in mice from 8 to 24 weeks of age on a high‐fat diet (HD) compared to the control group on a normal diet (CD) (Figure 1d). To further validate this finding, we measured hepatic *Oaf* mRNA levels in genetic mouse models. Consistent with the dietary model, *Oaf* mRNA was downregulated in the liver of male *ob/ob* mice and *db/db* mice compared to their age‐matched wild‐type controls (Figure 1e). This downregulation was also recapitulated in vitro; treatment of HepG2 cells with a mixture of oleic acid and palmitic acid (OA/PA) to mimic lipid overload led to a significant decrease in *Oaf* mRNA levels (Figure 1f). MASLD is characterized by elevated triglyceride (TG), total cholesterol (TCHO) and low‐density lipoprotein cholesterol (LDL‐C) levels in both liver and serum. To assess its clinical relevance, we examined the relationship between serum OAF concentrations and these lipid parameters. Correlation analysis showed that serum OAF levels were significantly negatively associated with both TG and TCHO levels in human clinical samples (Figure 1g). Furthermore, we found an inverse relationship between serum OAF levels and patient body mass index (BMI) by ELISA (Figure 1h). Collectively, these findings from mouse models and human subjects indicate that OAF is closely associated with metabolic dysregulation.

### 2.2 *Oaf* Deficiency Aggravates HD‐Induced Liver Steatosis

**FIGURE 2:**
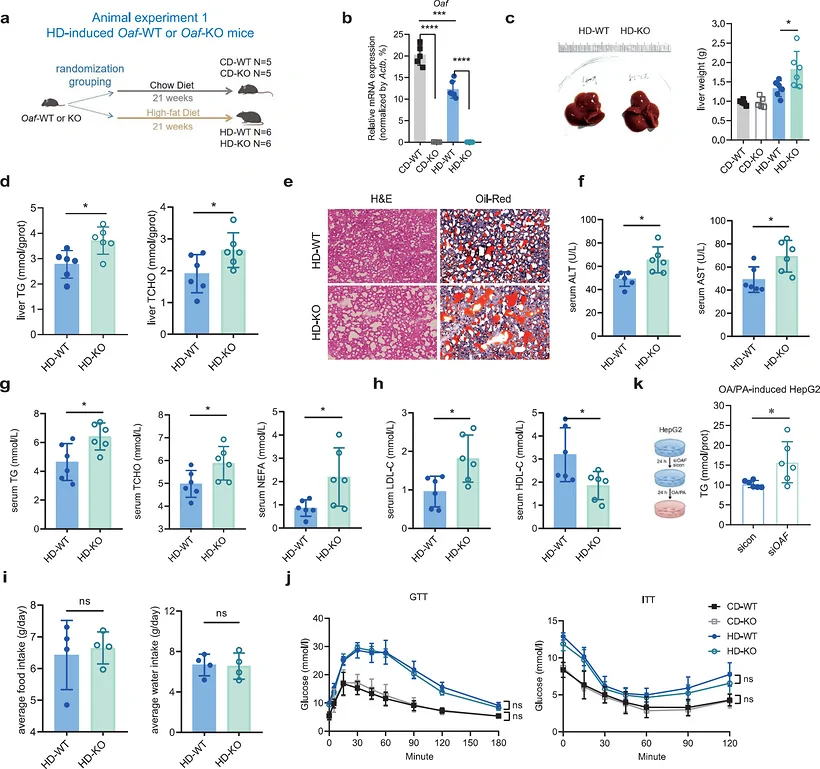
Depletion of *Oaf* exacerbates MASLD symptoms. (a–h) Seven‐week‐old male *Oaf*‐WT and *Oaf*‐KO mouse were fed either a CD or HD for 21 weeks and then sacrificed for analysis (N = 5 or 6 for each group), study design (a) and qPCR analysis *Oaf* mRNA expression in livers from four groups of mice (b). (c) Representative images of livers and liver weights from mice fed HD for 21 weeks. (e–f) Hepatic TG and TCHO concentrations (d) and representative images of H&E and Oil red O staining of liver sections from the two HD‐fed groups at the endpoint, scale bar = 50 µm (e). (f–h) Serum ALT and AST levels (f), TG, TCHO, and NEFA (g) as well as LDL‐C and HDL‐C (h) of the two HD‐fed groups. (i) Daily average food and water intake of mice monitored in metabolic cages during three consecutive days after HD‐feeding for 16 weeks, N = 4 for each group. (j) GTT and ITT results in four groups. (k) HepG2 cells transfected with si*OAF* or control siRNA for 36 h, followed by incubation with OA/PA medium for 12 h, intracellular TG level were then measured. Data were obtained from independent samples and analyzed using GraphPad prism 9. Data are presented as mean ± SD. (j) was analyzed by two‐way ANOVA, all others by two‐tailed unpaired student's *t*‐test. ^*^ *p* < 0.05; ^***^ *p* < 0.001; ^****^ *p* < 0.0001, “ns” indicates notsignificant. Statistical analysis by two‐tailed unpaired Student's *t*‐test.

To further investigate the function of OAF, we generated an *Oaf* knockout (*Oaf*‐KO) mouse model (Figure S3a). Seven‐week‐old male *Oaf*‐WT (wild‐type) and *Oaf*‐KO (knockout) mice were assigned to different dietary groups and fed either a standard chow diet (CD) or a HD for 21 weeks (Figure 2a). At the end of the feeding period, mice were sacrificed and hepatic *Oaf* mRNA levels were assessed (Figure 2b). Consistent with the previous results (Figure 1d), hepatic *Oaf* expression in HD‐fed WT mice was significantly lower than that in CD‐fed WT mice (Figure 2b). Livers from HD‐fed KO mice were increased in weight (Figure 2c) with elevated hepatic TG and TCHO levels (Figure 2d). Histological analyses revealed a pronounced increase in hepatic lipid accumulation in the HD‐KO group, as evidenced by the presence of more lipid droplets and enhanced Oil Red O staining (Figure 2e). In accordance with MASLD symptoms, serum levels of alanine aminotransferase (ALT), aspartate aminotransferase (AST), TG, TCHO, non‐esterified fatty acids (NEFA) and LDL‐C were also significantly elevated in HD‐fed KO mice, while high‐density lipoprotein cholesterol (HDL‐C) was correspondingly decreased (Figure 2f–h). This effect was further confirmed in HepG2 cells treated with OA/PA, where intracellular TG levels were notably higher in the si*OAF* group compared to controls (Figure 2k, Figure S4j). In addition, we investigated whether OAF might influence food intake and glucose metabolism. We used metabolic cages to measure mouse food and water intake during three consecutive days after HD‐feeding for 16 weeks, where we failed to detect differences between the two HD groups (Figure 2i). Glucose tolerance test (GTT) and insulin tolerance test (ITT) revealed that *Oaf‐*deficiency had no effect on mouse blood glucose levels or insulin sensitivity (Figure 2j). Given the possibility that OAF may also be produced or secreted by extrahepatic tissues, we examined *Oaf* mRNA expression across multiple tissues in obese and non‐obese WT mice under identical experimental conditions. The results showed that obesity did not alter *Oaf* mRNA expression in extrahepatic tissues (Figure S2d). To further investigate the contribution of hepatic OAF, we generated liver‐specific *Oaf* knockdown mice using an shRNA‐expressing adeno‐associated virus (shAAV) and subjected them to the same HD‐induced obesity protocol. We then evaluated the effects of hepatic *Oaf* silencing on hepatic lipid accumulation and other MASLD‐related phenotypes. Liver‐specific *Oaf* knockdown resulted in increased hepatic lipid accumulation, while changes in plasma TG, TCHO, LDL‐C, and HDL‐C levels were consistent with those observed in *Oaf* knockout mice (Figure S9). Collectively, these findings indicate that the OAF regulating MASLD‐related phenotypes is predominantly derived from the liver rather than from extrahepatic tissues. Taken together, these results demonstrate that *Oaf* deficiency exacerbates lipid accumulation and metabolic dysfunction in liver without affecting glucose metabolism or food intake.

### 2.3 OAF Supplementation Suppresses Hepatic Lipid Accumulation

**FIGURE 3:**
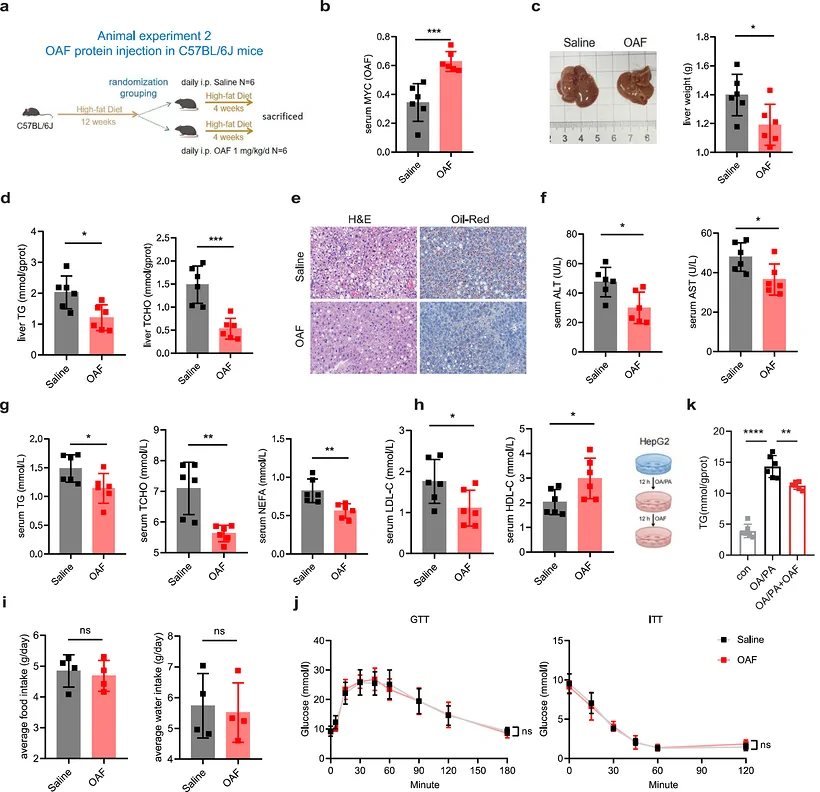
OAF supplementation reduces MASLD development. (a–h) HD‐fed C57BL/6J mice randomly divided into two groups receiving either OAF (1 mg/kg/day) or saline injections for 28 days (N = 6 for each group). Study design (a) ELISA analysis of serum OAF concentrations with MYC tag. (b). (c) Representative images of livers and liver weights from two groups of mice. (d, e) Hepatic TG and TCHO concentrations (d) and representative images of H&E and Oil red O staining of liver sections from the two groups at the endpoint, scale bar = 50 µm (e). (f–h) Serum ALT and AST levels (f), TG, TCHO, and NEFA (g) as well as LDL‐C and HDL‐C (h). (i) Daily average food and water intake of mice monitored in metabolic cages during three consecutive days in the final week, N = 4 for each group. (j) GTT and ITT results in two groups. (k) HepG2 cells were treated with OA/PA medium and 200 ng of OAF was added simultaneously. Intracellular TG levels were then measured. Data were obtained from independent samples and analyzed using GraphPad prism 9. Data were presented as mean ± SD. (j) was analyzed by two‐way ANOVA, all others by two‐tailed unpaired student's *t*‐test. ^*^ *p* < 0.05; ^**^ *p* < 0.01; ^***^ *p* < 0.001; ^****^ *p* < 0.0001, “ns” indicates not significant. Statistical analysis by two‐tailed unpaired Student's *t*‐test.

Given that OAF is a secreted protein, we next focused on evaluating the therapeutic potential of recombinant OAF in MASLD. Recombinant mouse OAF protein was intraperitoneally injected into HD‐fed C57BL/6J mice. Following 12 weeks of HD‐feeding, mice received daily injections of recombinant OAF for 28 consecutive days, with continued HD‐feeding throughout the treatment period (Figure 3a). OAF supplementation ameliorated the development of MASLD in C57BL/6J mice as reflected by reductions in liver steatosis (Figure 3b–e). In particular, liver weight was significantly reduced (Figure 3c), accompanied by decreased hepatic concentrations of TG and total TCHO (Figure 3d). Histological analyses using H&E and Oil Red staining also demonstrated that OAF administration reduced excessive lipid accumulation in the liver (Figure 3e). Serum levels of ALT, AST, TG, TCHO, NEFA or LDL‐C were also reduced after OAF injection, and HDL‐C was increased (Figure 3f–h). Once more, in vitro supplementation of recombinant OAF in OA/PA‐induced HepG2 cells markedly reduced intracellular TG accumulation (Figure 3k, Figure S4j). Consistent results were obtained in other OAF overexpression mouse models. Both OAF injection into *Oaf*‐KO mice and *Oaf* overexpression by using AAV*‐Oaf* significantly inhibited the development of MASLD (Figure S4a–d, f–i). It should be noted that, the inhibitory effect of OAF on MASLD is mediated by an intrahepatic mechanism rather than inter‐tissue interactions. This conclusion is supported by the observation that following AAV‐*Oaf* injection, *Oaf* mRNA expression increased exclusively in the liver, with no elevation detected in other tissues such as white or brown adipose tissue (Figure S4g). Furthermore, we compared the microscopic morphology of heart, lung and kidney tissue from two groups using H&E staining in OAF injection into *Oaf*‐KO mice experiments, we did not detect notable histological changes in the organization (Figure S4e). Meanwhile, we observed no obvious adverse health effects during the entire duration of the experiment (injection i. p. OAF with 1 mg/kg/day). Furthermore, GTT, ITT or food/water intake did not differ between OAF‐injected and control C57BL/6J mice (Figure 3i,j). Collectively, these results showed that OAF supplementation effectively attenuates hepatic lipid deposition, highlighting its potential as a metabolic regulator in the context of MASLD.

### 2.4 OAF Mediates Hepatic Metabolic Processes by Binding to SCPX

**FIGURE 4:**
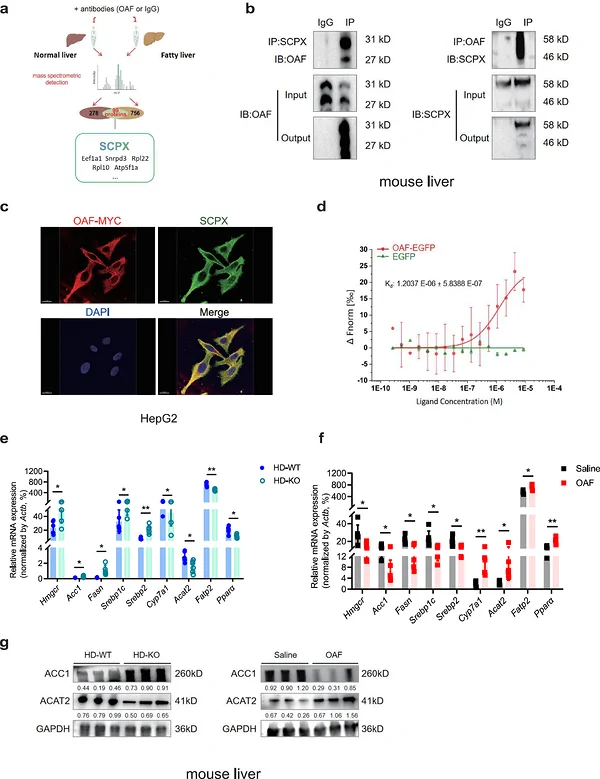
OAF reduces MASLD via SCPX and SCP2. (a) Schematic diagram of the Co‐IP and mass spectrometry screening workflow. Proteins in the box represent prominent proteins that were precipitated with OAF antibody, identified in both normal and fatty liver tissues. (b) Liver lysates from C57BL/6J mice were incubated with either OAF or SCPX antibodies, followed by a pull‐down assay. The interaction between OAF and SCPX was analyzed by Western blot. (c) HepG2 cells were supplemented with 200 ng OAF per well for 12 h. Immunofluorescence showed colocalization of MYC (OAF) and SCPX in HepG2 cells, scale bar = 15 µm. (d) Microscale thermophoresis demonstrated a direct interaction between OAF and SCPX (5 min) in lysates from HEK293T cells expressing EGFP‐OAF (N = 4). (e, f) qPCR to quantify the gene expression in liver samples from the HD‐fed model (N = 6 per group) (e) and the OAF injection model (N = 6 per group) (f). (g) Western blot analysis of ACC1 and ACAT2 protein expression in livers from HD‐fed and OAF injection mice; GAPDH was used as loading control. Data were obtained from independent samples and analyzed using GraphPad prism 9. Data are presented as mean ± SD. ^*^ *p* < 0.05; ^**^ *p* < 0.01; ^***^ *p* < 0.001. Statistical analysis was performed using a two‐tailed unpaired Student's *t*‐test.

To investigate the underlying mechanism by which OAF regulates hepatic metabolism, we performed Co‐immunoprecipitation (Co–IP) combined with mass spectrometry (MS) using anti‐OAF antibodies on total liver protein extracts from both normal and obese mice (Figure 4a). Among the identified interacting proteins, SCPX exhibited a strong interaction with OAF under both physiological and steatotic conditions (Figure 4a, Figure S5a). This interaction was confirmed by Co‐IP experiment and confocal microscopy, which demonstrated tight coupling and colocalization of MYC‐tagged OAF with SCPX (Figure 4b,c). MicroScale Thermophoresis (MST) analysis further verified the direct binding between OAF with SCPX (Figure 4d, Figure S5b,c), also we visualized the binding model between OAF and SCPX via Alphafold3 (Figure S5d). Furthermore, we examined the expression of genes previously reported to be regulated by SCPX or involved in lipid metabolism, including hepatic mRNA levels of 3‐hydroxy‐3‐methylglutaryl coenzyme A reductase (*Hmgcr*), acetyl coenzyme A carboxylase 1 (*Acc1*), fatty acid synthase (*Fas*), sterol regulatory element‐binding transcription factor 1c and 2 (*Srebp1c* and *Srebp2*), cytochrome P450 family 7 subfamily A member 1 (*Cyp7a1*), cholesterol acyltransferase 2 (*Acat2*), fatty acid transport protein 2 (*Fatp2*) and peroxisome proliferator‐activated receptor alpha (*Pparα*) [21, 23, 28]. These genes exhibited significant differential expression between the *Oaf*‐KO and WT groups (Figure 4e); *Acc1* mRNA (Figure 4e) or ACC1 protein levels increased and *Acat2*/ACAT2 decreased in KO vs. WT mice (Figure 4g). Consistently, treatment with recombinant OAF protein led to reduced serum TG, TCHO, and NEFA levels, accompanied by decreased *Acc1*/ACC1 mRNA/protein levels and increased *Acat2*/ACAT2 in OAF‐injected vs. saline injected mice (Figure 4f,g). These results support that OAF acts as a mediator of the metabolic process by interacting with SCPX, thereby inhibiting the progression of MASLD.

### 2.5 OAF Protects SCPX by Direct Binding and Inhibition of SIAH1‐Mediated Ubiquitination

**FIGURE 5:**
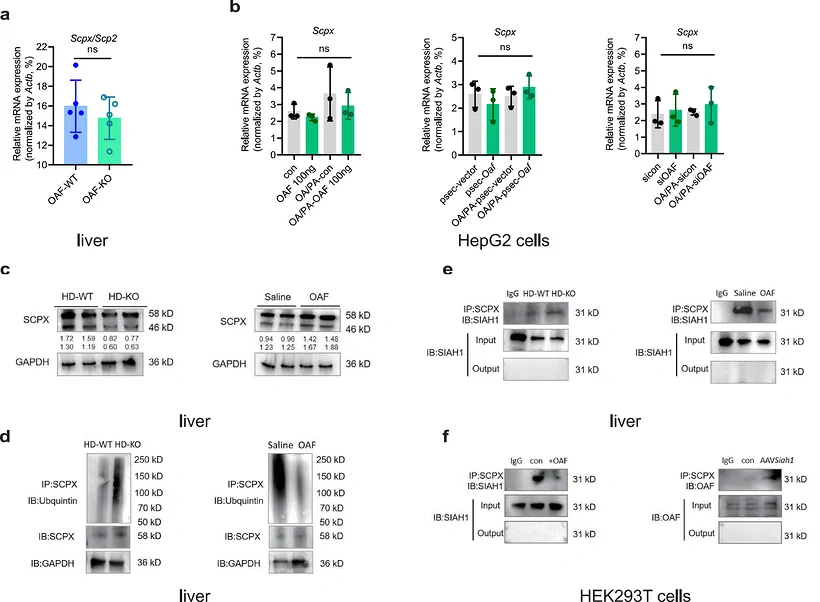
OAF promotes lipid metabolism by inhibiting SCPX ubiquitination. (a, b) OAF does not affect *Scpx* gene transcription. (a) qPCR analysis of *Scpx* mRNA expression in the livers of *Oaf*‐WT or *Oaf*‐KO mice (N = 5 per group). (b) *Scpx* mRNA expression in HepG2 cells following OAF supplementation, *Oaf* overexpression, or *Oaf* knockdown (N = 3 per group). (c) Western blot analysis of SCPX protein expression in livers from HD‐fed *Oaf*‐WT and *Oaf*‐KO mice, as well as from HD‐fed C57BL/6L mice with or without OAF treatment. Values represent the grayscale ratio of the respective bands to the corresponding GAPDH signal. (d, e) Liver lysates from HD‐fed *Oaf*‐WT and *Oaf*‐KO mice or HD‐fed C57BL/6L mice with or without OAF treatment were incubated with SCPX antibodies, followed by a pull‐down assay. Subsequently, Western blots were executed to detect the ubiquitination of SCPX (d), as well as the interaction between SCPX and SIAH1 (e). (f) HEK293T cell lysates supplemented with OAF or overexpressing SIAH1 were incubated with SCPX antibodies, followed by a pull‐down assay and Western blot analysis. Data were obtained from independent samples and analyzed using GraphPad prism 9. Data are presented as mean ± SD and analyzed by two‐tailed unpaired Student's *t*‐test, “ns” indicates not significant.

In mammalian livers, we found that OAF had no effect on the gene expression of *SCPX* in both in vitro and in vivo studies (Figure 5a,b, Figure S3b). However, *Oaf* deficiency resulted in reduced SCPX levels (both 58 kD and 46 kD) in liver under HD conditions, whereas intraperitoneal injection of recombinant OAF increased these two forms mentioned above in liver normalized to grayscale ratio of GAPDH loading control in Western blots (Figure 5c). SIAH1 is a conserved E3 ligase that ubiquitinates SCPX, promoting MASLD development [28]. We hypothesized that OAF might increase SCPX levels by inhibiting this ubiquitin‐dependent degradation. Supporting this hypothesis, SCPX ubiquitination was increased in HD‐fed *Oaf*‐KO mice but decreased upon OAF injection in C57BL/6J mice (Figure 5d). We further detected SCPX‐SIAH1 binding in liver lysates, consistent with our hypothesis, that the binding was enhanced in *Oaf*‐KO mice and weakened in upon OAF injection (Figure 5e). In vitro Co‐IP results revealed that the binding between SCPX and SIAH1 was suppressed upon OAF protein supplementation. In contrast, overexpression of SIAH1 failed to weaken the interaction between SCPX and OAF in HEK293T cells, confirming that OAF inhibits SCPX binding to SIAH1 (Figure 5f). Taken together, these results demonstrate that OAF protects SCPX from SIAH1‐mediated ubiquitination and degradation.

### 2.6 OAF Enters Hepatocytes via Endocytosis

To investigate how exogenous OAF enters cells, we treated HEK293T and HepG2 cells with endocytosis inhibitors, including chlorpromazine (CPZ) and methyl‐β‐cyclodextrin (MβCD). Immunofluorescence analysis revealed robust cytoplasmic accumulation of OAF in control cells, whereas OAF signals were undetectable in CPZ‐ or MβCD‐pretreated cells, indicating endocytosis as the primary uptake route (Figure S6a). Typically, internalized cargos are either delivered to lysosomes for degradation or recycled back to the plasma membrane. We therefore tested the effect of the lysosomal inhibitor bafilomycin A1 (Baf A1). OAF levels were slightly elevated after Baf A1 treatment (Figure S6b). Together, these findings indicate that OAF enters hepatocytes through endocytosis.

### 2.7 OAF‐Mediated Amelioration of MASLD Via SCPX

**FIGURE 6:**
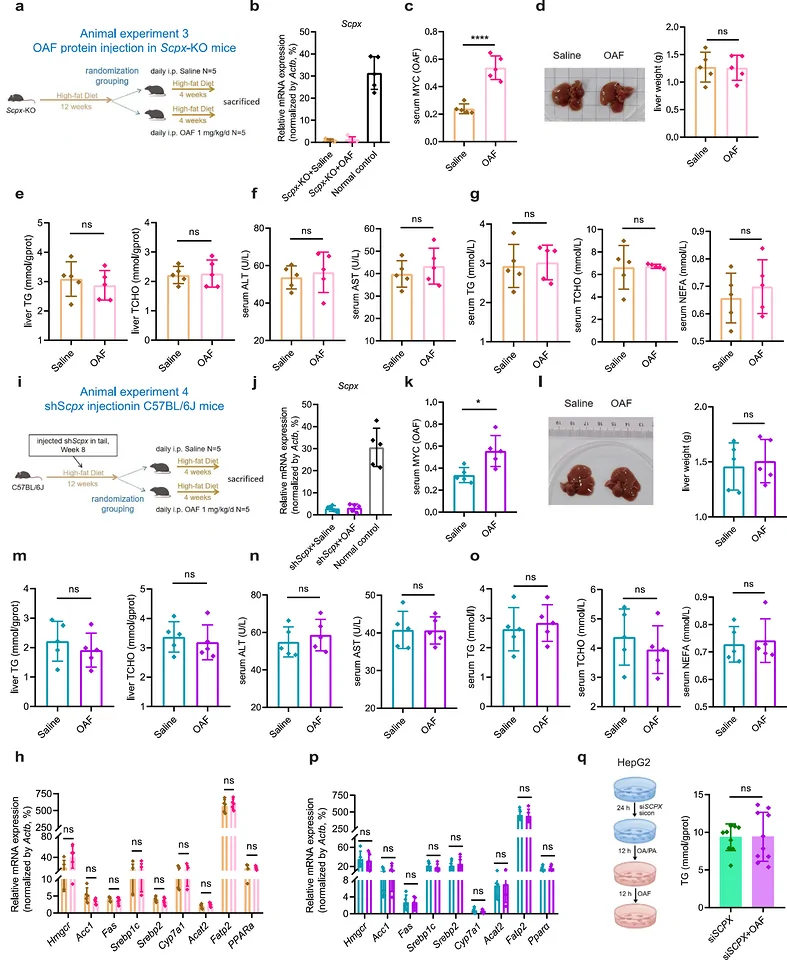
OAF has no effect on MASLD in *Scpx‐*KO and knockdown mice. (a) Eight‐week‐old male *Scpx*‐KO mice were fed a HD for 12 weeks, then randomly divided into two groups receiving either OAF (1 mg/kg/day) or saline injections for 28 days. Mice were sacrificed after a total of 16 weeks daily injections (N = 5 for each group). (b) qPCR analysis of *Scpx* mRNA expression in livers of the two groups. (c) ELISA measurement of serum OAF concentrations with MYC tag. (d) Representative images of livers and liver weights from two groups of mice. (e) Hepatic TG and TCHO concentrations in two groups at the endpoint. (f, g) Serum ALT and AST levels (f) as well as TG, TCHO, and NEFA (g) in two groups. (h) Quantification of *Hmgcr*, *Acc1*, *Fas*, *Srebp1c, Srebp2, Cyp7a1*, *Acat2, Fatp2* and *Pparα* gene expression in the liver. (i) Eight‐week‐old male C57BL6/J mice were HD‐fed for 12 weeks. At week 4 of HD‐feeding, mice received tail vein injection of AAV‐sh*Scpx*. At week 12, mice were randomly divided into two groups receiving either OAF (1 mg/kg/day) or saline injections for 28 days. Mice were sacrificed after a total 16 weeks of HD‐feeding (N = 5 for each group). (j) qPCR analysis of *Scpx* mRNA expression in livers of the two groups. (k) ELISA measurement of serum OAF concentrations with MYC tag. (l) Representative images of livers and liver weights from two groups of mice. (m) Hepatic TG and TCHO concentrations in two groups at the endpoint. (n, o) Serum ALT and AST levels (n) as well as TG, TCHO, and NEFA (o) in two groups. (p) Quantification *Hmgcr*, *Acc1*, *Fas*, *Srebp1c, Srebp2, Cyp7a1*, *Acat2, Fatp2* and *Pparα* expression in the liver. (q) Adipogenic assay in HepG2 cells. Intracellular TG level were measured. After transfection of HepG2 cells with si control (sicon) or si*SCPX* followed by OA/PA induction and OAF (200 ng) supplementation. Data were obtained from three independent samples and analyzed using GraphPad prism 9. Data are presented as mean ± SD and analyzed by two‐tailed unpaired Student's *t*‐test, “ns” indicates not significant.

To investigate the essential role of SCPX in mediating the metabolic effects of OAF on MASLD, *Scpx* knock‐out mice (*Scpx*‐KO) (Figure 6a,b, Figure S8a,b) were fed a HD for 12 weeks. As expected, OAF protein injections failed to reduce liver weight in HD‐fed *Scpx*‐KO mice (Figure 6c,d). The two groups also exhibited comparable liver TG and TCHO levels, as well as similar serum concentrations of ALT, AST, TG, TCHO and NEFA (Figure 6e–g). Furthermore, hepatic mRNA levels of *Hmgcr*, *Acc1*, *Fas*, *Srebp1c*, *Srebp2*, *Cyp7a1*, *Acat2, Fatp2* and *Pparα* did not differ between the groups (Figure 6h). Similarly, OAF protein failed to improve MASLD symptoms in C57BL/6J mice with liver‐specific *Scpx* knockdown (achieved by sh*Scpx* injection), mirroring the effects observed in C57BL/6J mice (Figure 6i–p). Finally, transfection of si*Scpx* into HepG2 cells abolished the TG‐lowering effect of OAF under OA/PA‐induced lipid accumulation (Figure 6q, Figure S8h). These results jointly illustrated that SCPX is a critical mediator of OAF's biological function in regulating liver fat accumulation.

## 3 Discussion

**FIGURE 7:**
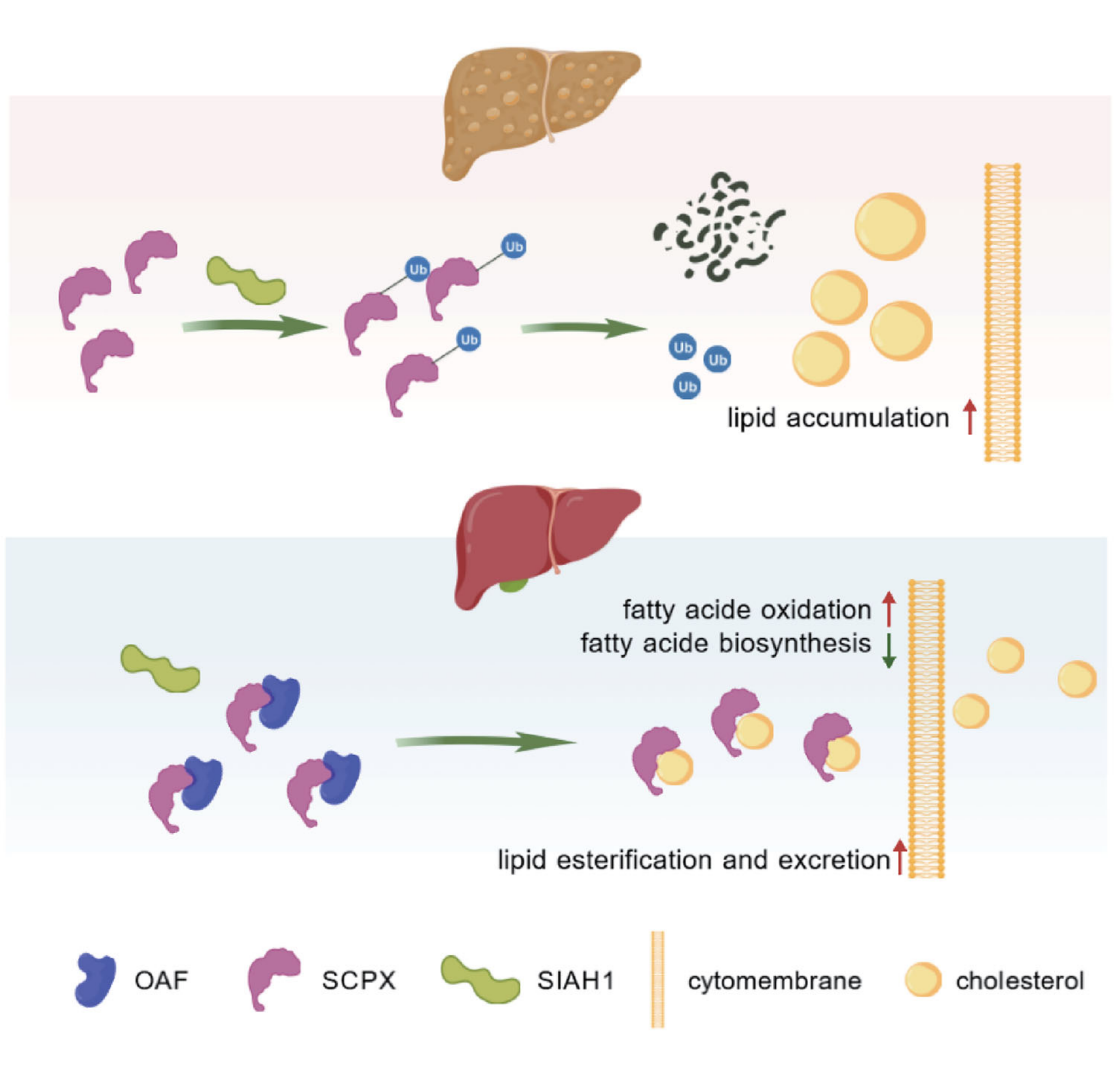
Proposed mechanism of OAF in MASLD. OAF is a novel protective factor in MASLD. The underlying mechanism is that OAF directly binds to and stabilizes the SCPX protein, preventing it from ubiquitin‐dependent degradation by the E3 ligase SIAH1. The accumulation of SCPX enhances the liver's capacity for lipid esterification and excretion, while promoting fatty acid oxidation and inhibiting de novo lipogenesis, ultimately alleviating the pathological phenotype of MASLD. This finding establishes the therapeutic value of OAF as a chaperone protein for SCPX.

The *Oaf* (out at first) gene was first identified in *Drosophila melanogaster* in 1995 [12]. Its protein sequence is highly conserved across animal phyla (Figure S1), yet its physiological role in mammals remains largely unexplored. Here, we show that *Oaf* expression is negatively correlated with serum lipid profiles in both mice and humans (Figure 1). *Oaf*‐deficiency aggravated MASLD symptoms in mice, whereas intraperitoneal administration of recombinant mouse OAF protein markedly suppressed disease development (Figures 2 and 3). Through a combination of in vivo and in vitro experiments, we demonstrate that OAF acts as a chaperone to inhibit MASLD. Mechanistically, OAF blocks the binding between SCPX and the E3 ligase SIAH1, thereby preventing SIAH1‐mediated ubiquitination and degradation of SCPX. This facilitates cholesterol esterification and excretion while regulating triglyceride metabolism (Figure 7). This discovery highlights OAF as a fundamentally new regulator of metabolic homeostasis and a potential therapeutic target for MASLD.

Molecular chaperones are fundamental guardians of proteostasis, enabling correct protein folding, localization, and turnover, while their dysfunction is intimately linked to disease pathogenesis, highlighting their therapeutic potential [29, 30]. Among various chaperone families, the BRICHOS superfamily has garnered particular interest due to its association with diverse human diseases, including respiratory distress, dementia, cancer, and COVID‐19 [31]. Advances in genomic sequencing and annotation have led to the identification of numerous BRICHOS‐containing proteins across 11 metazoan phyla, from worms and fish to humans [32]. Structurally, most BRICHOS proproteins are predicted or confirmed to be type II transmembrane or secretory proteins. They typically share a conserved architecture: an N‐terminal cytosolic domain, followed by a hydrophobic transmembrane region or signal peptide, a linker region, the defining BRICHOS domain, and a C‐terminal domain with high β‐strand propensity [31]. Recent bioinformatic analyses using human BRICHOS domain cores to query structural databases such as the AlphaFold human proteome have further expanded and refined this family. Notably, through such approaches, OAF has been identified as a novel member of the human BRICHOS chaperone family [19]. However, there is a lack of specific experimental validation. In the present study, we show that OAF binds to and enhances SCPX stability by preventing its SIAH1‐mediated ubiquitination and degradation, thereby functioning as a molecular chaperone. This mechanism parallels that of another hepatic secretory chaperone, mesencephalic astrocyte‐derived neurotrophic factor (MANF), which competitively binds to calcium‐binding proteins S100A8/9 to regulate hepatic fibrosis by interrupting TLR4 signaling [33]. Our results provide direct experimental validation for the prediction by Sanchez‐Pulido et al. that OAF is a member of the BRICHOS chaperone family. Furthermore, our co‐immunoprecipitation assays identified several candidate binding partners for OAF (Figure 4a). We hypothesize that OAF might exert its chaperone function by interacting with these proteins and modulating their activity, a possibility that merits future exploration.

Both data from the Human Protein Atlas and our experimental results confirm that OAF is a secreted protein highly expressed in the liver (Figure 1, Figure S2). The administration of purified, eukaryotic‐expressed OAF protein significantly ameliorated HD‐induced MASLD phenotypes, underscoring its potential therapeutic value. Using pharmacological inhibitors, we confirmed that OAF enters cells through endocytosis (Figure S6a). The physiological rationale for this autocrine signaling mechanism remains unclear. While numerous liver‐secreted factors are closely related to metabolic disturbance, such as fibroblast growth factor 21 (FGF21), glycoprotein nonmetastatic melanoma protein B (GPNMB) and angiopoietin‐like 3 (ANGPTL3), etc [34, 35, 36, 37, 38], yet, such liver‐derived factors utilized in an autocrine/paracrine manner are not common. Notable examples include arylsulfatase A (ARSA), which mediates MASLD progression via autocrine/paracrine signaling to alter local and systemic metabolism [39], and NETRIN1, which drives hepatic fibrosis through an autocrine loop in hepatic stellate cells via UNC5B receptors, triggering Ca^2^^+^ influx and downstream CAMKII‐SMAD2 signaling [40]. Given that OAF protein is also detectable in other tissues such as the kidney, colon, and lung, despite low corresponding mRNA levels, its secretion may primarily serve inter‐tissue and implies a role in inter‐organ signaling, potentially leveraged by the liver during lipid overload. However, our current results do not establish whether OAF must be secreted to exert its function within the liver, a possibility that warrants further investigation.

Our data demonstrate that global knockout or liver‐specific silencing of SCPX completely abolishes the therapeutic effect of OAF on MASLD, suggesting that the protective role of OAF in MASLD is primarily mediated through liver‐intrinsic mechanisms (Figure 6i–p). Due to the fact that MASLD is often associated with other metabolic disorders [2, 4, 41], we evaluated the impact of OAF on adiposity. While genetic ablation or supplementation of OAF did not yield statistically significant changes in mouse body weight, we observed non‐significant trends suggesting a moderate inhibitory effect on obesity (Figure S7). Notably, metabolic cage analyses revealed that OAF enhances whole‐body energy expenditure (Figure S7e,f). In contrast, deficiency in *SCPX* did not affect body weight in obese mice although it undoubtedly possesses lipid transport capability (Figure S8c), implying that OAF's metabolic effects may be independent of this pathway. The mechanism underlying OAF's influence on energy balance warrants further investigation, potentially through studies using tissue‐specific knockout models.

In this study, we detected OAF protein in other organs, including the kidney, lung, and intestine, raising the possibility that OAF may regulate physiological functions beyond the liver (Figure S2). Hepatic *Oaf* mRNA levels were significantly higher than those in the kidney, whereas protein levels were comparable between the two tissues (Figure S2b), suggesting potential differences in OAF translation efficiency or post‐transcriptional regulation. Furthermore, AAV‐mediated *Oaf* overexpression markedly increased circulating OAF levels, whereas shAAV‐mediated *Oaf* silencing significantly reduced them (Figure S10c,d). These findings indicate that hepatic OAF can be secreted into the circulation and that its hepatic expression positively correlates with plasma OAF protein levels. Notably, a recent study confirmed that the liver‐derived hormone fibrinogen‐like protein 1 (FGL1) acts as a key mediator of liver‐kidney crosstalk in renal fibrosis [42]. Together, these findings suggest that OAF, like FGL1, may be secreted by the liver and act on the kidney‐an intriguing possibility that merits further study. However, this hypothesis remains speculative and requires further experimental validation.

To preliminarily assess the therapeutic potential of OAF, we determined its plasma concentration profile and estimated its circulating half‐life in rats following intraperitoneal administration, according to established protocols for pharmacokinetic analysis [43, 44]. Following a single intraperitoneal injection (0.5 mg/kg), plasma OAF levels peaked at approximately 2‐ to 3‐fold above the physiological baseline, with an estimated half‐life of ∼12 h and near‐complete clearance within 24 h after administration (Figure S10a). In a separate experiment, daily intraperitoneal administration of OAF (1 mg/kg/day) for 8 consecutive days in mice did not result in detectable plasma accumulation (Figure S10b), consistent with its relatively short circulating half‐life. These findings indicate that OAF is rapidly cleared in vivo and exhibits minimal accumulation upon repeated dosing, providing preliminary pharmacokinetic evidence supporting its potential development as a therapeutic agent. Nevertheless, further studies are required to comprehensively evaluate its long‐term safety, immunogenicity, pharmacodynamic characteristics, and therapeutic efficacy.

In conclusion, our work identifies OAF as a previously uncharacterized molecular chaperone that safeguards hepatic lipid metabolism and metabolic health. While further research is needed to fully elucidate its mechanisms, this finding underscores its potential as a novel therapeutic target, offering a promising avenue for drug development against MASLD and related metabolic diseases.

## 4 Materials and Methods

### 4.1 Plasmids and Small Interfering RNA Construction

Mouse *Oaf* sequence was reverse‐transcribed from hepatic mRNA and subsequently cloned into the pSec‐His/Myc expression vectors, respectively. pcDNA3.1‐*Oaf*‐EGFP, pcDNA3.1‐EGFP plasmids, primers and small interfering RNAs were synthesized by General Bio Co., Ltd (Anhui, China). pLV3‐CMV‐*Siah1*(mouse)‐3 × FLAG‐CopGFP‐Puro plasmid was purchased from miaolingbio Co., Ltd (Wuhan, China). The sequences of small interfering RNAs are listed in Table S1.

### 4.2 Cell Experiments

HepG2, CHO and HEK293T cells were obtained from the American Type Culture Collection (ATCC). Cells were cultured in DMEM (Gibco) supplemented with 10% fetal bovine serum (FBS, Gibco) and 1% Penicillin‐streptomycin (CM‐0001, Sparkjade), referred to as regular medium, in a 37°C incubator with 5% CO_2_. Liposomal Transfection Reagent (40802ES, Yeasen) was diluted in DMEM and replaced with regular medium after 6 to 8 h. Oleic acid (OA) and palmitate (PA) were purchased from Sigma (O1008 & P0500) and diluted to final concentrations of 0.66 and 0.33 mm, respectively, in DMEM containing 2% BSA (A8010, Solarbio). For inhibiting the activity of lysosomes, Bafilomycin A1 (Baf A1, 100 nm, MedChemExpress, HY‐00558) was added 6 h prior to OAF (200 ng) supplementation. To evaluate the binding between SCPX and SIAH1, binding experiments were conducted as follows: HEK293T cells were starved for 12 h prior to OAF supplementation; SIAH1 overexpression was achieved via liposome transfection as described above; and subsequent co‐IP and Western blot analyses were performed.

### 4.3 Immunofluorescence

Cells were seeded onto glass coverslips placed in a 12‐well plate at an appropriate density. After adherence, the cells were fixed with 4% PFA, permeabilized with Triton X‐100, and blocked with 5% BSA for 1 h at room temperature. Subsequently, the cells were co‐incubated overnight with antibodies. On the following day, cells were incubated with the appropriate fluorophore‐conjugated secondary antibodies for 2 h at room temperature in the dark. Nuclei were then stained with DAPI for 5 min under light‐protected conditions. Finally, the coverslips were mounted using an anti‐fade mounting medium, and fluorescence images were acquired using a fluorescence microscope. In endocytosis inhibition experiment, cells were pretreated with chlorpromazine (CPZ, 100 µM, MedChemExpress, HY‐12708) or methyl‐β‐cyclodextrin (MβCD, 100 mg/mL, MedChemExpress, HY‐101461) one hour prior to OAF (200 ng) supplementation. All antibodies used are listed in Table S2.

### 4.4 Recombinant OAF Protein Extraction

Recombinant OAF protein was purified from the conditioned medium of CHO cell lines that stably express mouse *Oaf*. The stable cell line was established by transfecting CHO cells with pSec‐*Oaf‐*His/Myc for 36 h, followed by selection using 400 µg/ml Zeocin (R25001, Invitrogen) for 2 to 3 weeks.

For protein production, stably transfected cells were expanded in 15 cm culture dishes. Upon reaching confluence, the medium was replaced with serum‐free medium for a 60 h conditioning period. The conditioned medium was collected and filtered through a 0.45‐µm membrane before purification. OAF‐His/MYC protein was purified by nickel‐affinity chromatography using Sefinose 6FF resin (C600332, BBI). Protein concentration was determined using a BCA assay (T9300A, Takara), and the purified protein was aliquoted and stored at −80°C for future use.

### 4.5 Animal Management and Licensing

*Oaf* knockout mice (T034472), *Scpx* knockout mice (T028571), 16‐week‐old male *ob/ob* mice, 18–week‐old male *db/db* mice, C57BL/6J mice and Male Sprague‐Dawley (SD) rats (7‐week‐old, 250∼280 g) were obtained from Gempharmatech Co., Ltd (Nanjing, China). *Oaf* knockout mice were generated using CRISPR‐Cas9 technology by deleting the nucleotides of exon 2 and 3. *Scpx* knockout mice were also generated by deleting the nucleotides of exon 2 and 3, leading to subsequent frameshift mutations and loss of protein function. All mice were housed in a specific pathogen free (SPF) grade facility under a 12 h‐light / dark cycle. Mice were kept in plastic cages with fewer than five animals per cage and had free access to food and water. Twenty‐one‐day‐old male Chinese yellow‐feathered broilers were purchased from a local commercial hatchery. All animal care and experimental procedures were conducted in accordance with the Animal Management Regulations of the Ministry of Health, China (Document No. 55, 2001), and were approved by the Science and Technology Department of Jiangsu Province [SYXK (SU) 2020–0047].

### 4.6 Animal Experiments

#### 4.6.1 HD‐Feeding Experiment

A 60% High‐fat–diet (XTHF60) was purchased from Pharmaceutical Bioengineering Co., Ltd (Nanjing, China) and administered to designated groups according to the study design. For *Oaf‐*WT or ‐KO mice, a 60% high‐fat diet was provided throughout the entire experimental period without any additional experimental procedures; for all OAF injection experiments, recombinant OAF protein was intraperitoneally injected at a dose of 1 mg/kg/day for 28 consecutive days. The body weights were continuously recorded during the experiment as well as the final fat and liver weights, when sampling the tissues. Energy expenditure testing was executed during the final week in metabolic cages (N = 4).

#### 4.6.2 Glucose Tolerance Test (GTT)

GTT was performed before the mice were sacrificed. Briefly, mice were fasted for 16 h prior to i.p. injection of glucose (2 g/kg), and blood glucose levels were measured by collecting blood from the tail tip at 0, 30, 60, 90, 120, 150 and 180 min.

#### 4.6.3 Insulin Tolerance Test (ITT)

ITT was performed two weeks before the mice were sacrificed. Briefly, mice were fasted for 2 h prior to i.p. injection of insulin (0.8 U/kg), and blood glucose levels were measured by collecting blood from the tail tip at 0, 15, 30, 45, 60, 90 and 120 min. Insulin was purchased from Procell Co., Ltd (Wuhan, China, PB180432).

#### 4.6.4 Adeno‐Associated Virus Conduction

AAV‐control (pAAV‐CBh‐EGFP‐WPRE), AAV‐*Oaf* (pAAV‐CBh‐Oaf‐3xFLAG‐P2A‐EGFP‐WPRE), shAAV‐control (pscAAV‐U6‐shRNA(NC2)‐CMV‐EGFP‐tWPAE), shAAV‐*Scpx* (pscAAV‐U6‐shRNA(*Scpx*)‐CMV‐EGFP‐tWPA), and shAAV‐*Oaf* (pAAV‐U6‐shRNA(*Oaf*)‐CBh‐EGFP‐WPRE) vectors were designed and produced by OBiO Technology (Shanghai) Corp., Ltd. Viral particles were administered via tail vein injection at a dose of 2 × 10^11^ viral genomes (vg) per mouse, delivered in 100 µL sterile saline.

#### 4.6.5 Half‐Life Measurement

The circulating half‐life of OAF was determined in male SD rats. Following a single intraperitoneal injection of recombinant OAF protein (0.5 mg/kg), blood samples were collected at 0, 3, 6, 12, 18, and 24 h post‐injection. Serum was isolated by centrifugation, and OAF concentrations were quantified using ELISA. The circulating half‐life of OAF was estimated based on the serum concentration‐time profile.

### 4.7 Serum Analysis

Serum samples (both clinical and mouse specimens) were collected from whole blood after centrifugation. TG, TCHO, NEFA, AST, ALT, LDL‐C and HDL–C were detected using commercial assay kits according to the manufacturer's instructions (A042‐2‐1, A110‐1‐1, A111‐1‐1, C009‐2‐1, C010‐2‐1, A112‐1‐1, A113‐1‐1 Nanjing Jiancheng, China). ELISA test was executed to detect the OAF levels. In brief, 100 µL of serum was first processed with an albumin removal reagent (WA‐013, Invent), and then coated onto a 96‐well plate (C1055, Solarbio) at 37°C for 4 h. The plate was then blocked with 5% milk for 2 h and incubated with the OAF antibody (PA5‐89072, ThermoFisher) overnight at 4°C. On the following day, after incubation with HRP‐labeled goat anti‐rabbit IgG (A0208, Beyotime Biotechnology, Shanghai) for 2 h at 37°C, the optical density (OD) was measured at 370 nm using TMB Chromogen Solution (P0208, Beyotime Biotechnology, Shanghai).

### 4.8 Western Blot and Co‐Immunoprecipitation (Co‐IP)

Total proteins were extracted from tissues or cell lysates using RIPA buffer (PC101, Epizyme). Protein concentrations were determined using a BCA kit (T9300A, Takara). Equal amounts of protein were separated by SDS‐PAGE and transferred to PVDF membranes. The membranes were blocked with 5% milk for 1 h and soaked overnight at 4°C with various primary antibodies. On the following day, protein expression levels were assessed after incubation with HRP‐labeled goat anti‐rabbit or anti‐mouse secondary antibodies for 1 h.

Co‐IP was performed according to the manufacturer's instructions (P2179S, Beyotime Biotechnology, Shanghai, China). Briefly, after lysis of mouse liver tissue or cells, the target protein was bound to magnetic beads according to the instructions, and the bound proteins were detected by Western blot the following day. All antibodies used are listed in Table S2.

### 4.9 Real‐Time qPCR

Total RNA was extracted using RNAiso Plus (9109, Takara) and reverse‐transcribed into complementary DNA (cDNA) using the HiScript III RT SuperMix for qPCR kit (R323‐01, Vazyme, Nanjing, China). Quantitative PCR (qPCR) was operated on a LightCycler QuantStudio 5 (ThermoFisher) using SuperStar Universal SYBR Master Mix (CW3360, CoWin). Relative mRNA expression levels were calculated using the 2^−ΔΔCt^ method and normalized to the endogenous reference gene *Actb*. Primer sequences used for qPCR are listed in Table S3.

### 4.10 Histological Staining

H&E and Oil Red staining were performed using standard protocols. Briefly, livers and WAT tissues fixed in 4% paraformaldehyde (PFA) were cut into 5 µm‐thick slices, and routine H&E and Oil Red staining was performed by Nanjing Dreambio Co., Ltd (Nanjing, China).

### 4.11 Bioinformatics Analysis

Protein CDS sequence was downloaded from NCBI, and the amino acid sequences were aligned using Mega 11.0. Heatmap data in this study were obtained from GDS6248 in the Gene Expression Omnibus (GEO) repository. Our analysis were performed online using BioLadder (https://www.bioladder.cn) [45]. Protein sequences of OAF and SCPX were uploaded to https://alphafold3.org/ and docked to generate 3d structural models. Docking results were visualized using PyMOL 2.0.

### 4.12 Binding Protein Screening and Validation

Mass spectrometry (MS) analysis was performed by West China Hospital of Sichuan University. Normal and fatty liver tissues were homogenized, and equal amounts of OAF antibody or IgG control antibody were added separately to the supernatants after centrifugation. Co‐IP was then carried out following standard protocols. The next day, after rinsing the magnetic beads, samples were transported to the testing laboratory under −80°C conditions. MicroScale Thermophoresis (MST) analysis was conducted by Zoonbio Biotechnology Co., Ltd (Nanjing, China). HEK293T cells were transfected with either pcDNA3.1‐OAF‐EGFP or pcDNA3.1‐EGFP plasmids. After 48 h, the transfected cells were lysed and centrifuged, and the resulting supernatants were transported to the testing laboratory under −80°C conditions in a dark container.

### 4.13 Clinical Samples

Clinical samples were collected by West China Hospital, Sichuan University, with the approval of the local Ethical Committee and Review Board of West China Hospital (No. 2025[287]). The requirement for informed consent was waived by the Ethical Committee because the study used residual blood samples from patients that were collected after routine clinical testing. Information of the clinical samples is provided in Table S4.

### 4.14 Statistical Analysis

Data were taken from at least three independent samples and presented as means ± standard deviation (SD). For comparisons between two groups, a two‐tailed unpaired Student's *t*‐test was used for bar graph data. For longitudinal assessments, including body weight trajectories, GTT and ITT curves, a two‐way repeated‐measures ANOVA was applied to evaluate the effects of treatment, time, and their interaction, followed by post–hoc comparisons using Sidak's test. Correlation visualizations were generated using the ggcorrplot package based on ggplot2. Statistical significance was determined as *p* < 0.05, with significance levels indicated as follows: *p* < 0.05 (^*^); *p* < 0.01 (^**^); *p* < 0.001 (^***^); *p* < 0.0001 (^****^).

## Author Contributions

C.D. and S.L. conceived and supervised the whole project; Z.L., J.S., K.M., S.T., S.W., W.J., C.L., T.Z. and H.Z. conducted experiments; Z.L., J.S., K.M., W.J., X.Z, S.L. and C.D. performed data analysis and interpretation; Z.L and S.L wrote the paper; S.L and C.D. revised the paper.

## Conflicts of Interest

The authors declare no conflicts of interest.

## Supporting information




**Supporting File 1**: advs77451‐sup‐0001‐SuppMat.docx.

**Supporting File 2**: advs77451‐sup‐0001‐SuppMat.docx.

## Data Availability

The data that support the findings of this study are available in the supplementary material of this article.
