# Supplementary material for: OAF Blocks SIAH1‐Mediated Degradation of SCPX, a Therapeutic Strategy for MASLD
Source: Adv Sci (Weinh). 2026 Aug 29:e77451. Online ahead of print. doi: 10.1002/advs.77451 (PMC13525414; doi:10.1002/advs.77451)
Supplement: Supplementary file 1 [file ADVS-9999-e77451-s002.pdf]

ID	TC	TG	LDL	ID	TC	TG	LDL
4085	14.84	6.10	1.31	4423	7.58	5.65	4.47
4401	2.17	1.33	0.62	4027	2.41	1.19	1.07
4001	8.49	6.67	1.05	4003	3.48	0.64	2.30
4214	13.67	2.65	3.72	4106	2.80	0.49	1.65
4193	4.68	6.25	0.52	4677	6.26	1.47	4.52
4089	9.10	14.38	0.55	4649	6.27	1.98	4.53
4017	1.08	0.71	0.49	4011	7.34	7.74	3.54
4080	1.76	5.90	0.44	4458	6.24	1.69	4.32
4205	2.32	1.95	0.97	4009	3.00	0.78	1.62
4001	1.27	1.29	0.57	4307	2.25	1.15	0.80
4015	1.96	1.23	0.80	4635	7.70	2.13	5.59
4103	1.91	1.56	0.76	4207	2.64	0.53	1.18
4199	4.96	5.61	1.10	4091	6.63	1.41	4.87
4034	1.58	1.53	0.44	4108	6.99	1.66	5.17
4070	6.15	11.07	0.57	4011	6.83	6.10	4.08
4116	2.92	1.54	1.63	4010	3.61	0.99	1.68
4020	6.56	1.49	3.45	4043	6.34	1.53	4.55
4203	2.74	0.85	1.70	4306	3.57	0.81	1.93
4002	1.66	0.35	0.95	4283	7.07	1.03	5.31
4032	3.46	1.63	2.15	4026	3.34	0.56	1.62
4236	6.81	6.75	4.40	4007	4.25	0.77	2.36
4007	3.62	6.18	1.53	4308	4.39	0.43	2.66
4111	2.90	1.99	1.08	4414	7.52	1.04	5.29
4016	6.22	7.86	3.26	4046	6.32	0.75	3.91
4036	4.11	11.69	0.70	4612	6.60	0.61	3.92
4030	3.34	2.04	2.05	4234	6.73	2.54	2.33

gender	ID	BMI
famale	0039841601	14.9755278
famale	0038199785	16.9921875
famale	0039839633	18.02595738
famale	0039697671	18.04803554
famale	0039721871	18.22222222
famale	0000581571	18.28989483
famale	0039848087	19.22768787
famale	0038293839	19.43634597
famale	0003545948	19.51840894
male	0000087441	19.83471074
famale	0037661208	20.81165453
famale	0037497088	20.81165453
famale	0014822837	20.81165453
famale	0000147430	20.93212012
male	0038003775	20.95717116
famale	0039853331	21.33821064
male	0000984284	22.11043044
famale	0039845140	22.37034332
male	0039854608	22.49134948
male	0039850467	22.49134948
male	0039834464	22.67573696
famale	0020449477	23.36989217
male	0020006499	23.71184463
famale	0033839640	23.78121284
male	0039838805	23.87511478
famale	0000178764	24.13601606
male	0039855086	24.22145329

gender	ID	BMI
famale	001029723	24.24242424
male	003964269	24.33747972
famale	003985001	24.34960912
male	001877213	24.38652644
famale	003983137	24.43518667
male	003957356	24.76756592
male	003966440	24.76756592
male	003860131	24.8015873
famale	003985542	24.97398543
famale	003248974	25
male	003984594	25.03413746
famale	003985533	25.27120316
male	001644002	25.35154137
male	003984559	25.51020408
male	003984559	25.51020408
male	002061711	25.60553633
famale	001926038	25.88757396
male	003959196	26.57312925
male	003985206	26.57312925
famale	003984486	26.66666667
famale	001310687	27.51338489
male	002076414	27.63605442
male	003872948	28.73469388
famale	003887178	28.83506344
male	003980902	29.38475666
famale	003985090	29.57679827
male	003985088	30.09473
male	001991463	33.68587452

	ALT（U/L）	AST（U/L）	GLU（mmol/L）
TG>5.6	27.68 ± 22.41	30.83 ± 22.88	11.00 ± 6.35
TG<2	24.65 ± 20.09	37.10 ± 42.05	6.54 ± 2.12
TCHO>6.2	25.61 ± 19.62	30.7 ± 20.29	7.84 ± 1.41
TCHO<3.5	24.00 ± 13.07	32.35 ± 17.74	6.28 ± 1.54
