# Supplementary material for: OAF Blocks SIAH1‐Mediated Degradation of SCPX, a Therapeutic Strategy for MASLD
Source: Adv Sci (Weinh). 2026 Aug 29:e77451. Online ahead of print. doi: 10.1002/advs.77451 (PMC13525414; doi:10.1002/advs.77451)
Supplement: Supplementary file 2 [file ADVS-9999-e77451-s001.docx]

**OAF Blocks SIAH1-mediated Degradation of SCPX, a Therapeutic Strategy for MASLD**

**Supplementary figure legends:**

## Supplementary Figure 1 OAF is conserved over a wide range of animal species.

**a**-**b**. Amino acid sequence alignment across mammalian species (**a**) and representative taxonomic groups (**b**), using Mega 11. The predicted signal peptide region and BRICHOS domain are boxed in red and blue, respectively.

## Supplementary Figure 2 *Oaf* expression in C57BL/6J mouse model and chicken.

**a**-**b**. qPCR and Western Blot were used to measure *Oaf* mRNA expression (**a**) and protein levels (**b**) in various tissues of C57BL/6J mice at different ages (N = 4 per group). **c**. qPCR analysis *Oaf* mRNA expression in different tissues from 21-day-old broiler chickens (N = 4 per group). **d**. Seven-week-old male WT mice were fed either a CD (N = 6) or an HD (N = 5) for 21 weeks, followed by qPCR analysis of *Oaf* mRNA expression in various tissues. *, indicates compared to the column representing the liver and #, compared to the kidney. Statistical analysis by two-tailed unpaired Student’s t-test, “ns” indicates not significant.

## Supplementary Figure 3 Validation of *Oaf* knockout, overexpression and knockdown models.

**a.** CRISPR/Cas9 editing strategy used to generate frameshift deletions in *Oaf* by targeting exons 2 and 3. **b**. qPCR analysis of *OAF* mRNA in HepG2 cells after transfection with a psec‑*OAF* overexpression plasmid or *OAF*‑targeting siRNA (N = 6 per group). Statistical analysis by two-tailed unpaired Student’s t-test, ****, *P* < 0.001, “ns” indicates not significant.

## Supplementary Figure 4 OAF reduces MASLD by protein injection in *Oaf*-KO mice as well as in an AAV-*Oaf* overexpression model.

**a**. Seven-week-old male *Oaf*-KO mice were HD-fed for 12 weeks, then randomly divided into two groups receiving either OAF (1 mg/kg/day) or saline injections for 28 days. Mice were sacrificed after a total of 16 weeks HD-feeding (N =7 for each group). **b**. ELISA analysis of serum OAF concentrations with MYC tag at the endpoint. **c.** Representative images and liver weights at endpoint. **d**. Hepatic TG and TCHO concentrations of livers from mice fed an HD for 16 weeks. **e.** Representative images of H&E staining of heart, lung and kidney sections from the two groups, scale bar = 50 μm. **f**. Eight-week-old male C57BL6/J mice were randomly assigned to receive either AAV-control (AAV-con) or AAV-*Oaf* via tail-vein injection and were subsequently fed a high-fat diet (HD) for 15 weeks (n = 5 per group). **g.** *Oaf* mRNA expression assessed by qPCR in liver, BAT, iWAT and colon. **h.** Representative images and liver weights at the endpoint. **i**. Hepatic TG and TCHO concentrations in mouse livers at the endpoint. **h.** Representative images and body weight in mice from the two groups at the endpoint. **i.** Representative images of OA/PA-induced HepG2 cells after *OAF* knockdown or OAF protein supplementation (scale bar = 200 μm). Arrows indicate lipid droplet accumulation. Data were obtained from independent samples and analyzed using GraphPad prism 9. Data were presented as mean ± SD. ***, *P* < 0.05; **, *P* < 0.01; ***, *P* < 0.005, ****, *P* < 0.001. Statistical analysis by two-tailed unpaired Student’s t-test.

## Supplementary Figure 5 OAF directly binds to SCPX.

**a**. SCPX peptides identified by mass spectrometry after co-IP pull-down with OAF antibodies. Red letters represent peptides detected by mass spectrometry. The yellow highlight indicates the region corresponding to the 13 kD SCP2 moiety. **b-c**. Original MST fluorescence traces under varying concentrations, with normalized curves shown on the right. EGFP (**b**) and EGFP-tagged OAF (**c**). **d**. The predicted binding structures in mice using AlphaFold3 and visualized with PyMOL, OAF and SCPX, color code: yellow, OAF; green, SCPX; red, potential polar binds.

## Supplementary Figure 6 Exogenous OAF enters cells via endocytosis.

**a**. Immunofluorescence showing that endocytosis inhibitors block OAF uptake into HEK293T and HepG2 cells. **b**. Western blot analysis of OAF-MYC levels in HEK293T cells treated with lysosomal inhibitor bafilomycin A1 (Baf A1) followed by OAF (200 ng) supplementation.

## Supplementary Figure 7 OAF may slightly alleviate obesity symptoms accompanied by MASLD.

**a-b**. Representative images of mice at the endpoint and body weight over 21 weeks in *Oaf-*WT and -KO mice fed CD or HD (N = 5 or 6 per group) (**a**). BAT, total WAT weights and lean mass/body weight ratios at the endpoint (**b**). **c-d**. Representative images of mice at the endpoint and body weight over 28 days resulting from OAF protein injection in HD-fed C57BL/6J mice (N = 6 per group) (**c**). BAT, total WAT weights and lean mass/body weight at the end point (**d**). **e-f.** Energy expenditure (EE) in metabolic cages during the final week of HD-WT or KO mice (**e,** N = 4 for each group) and OAF protein injection in HD-fed C57BL/6J mice (**f,** N = 4 for each group), the right column represents the area under curve (AUC) of the EE (left). **g-h**. OAF injection into HD-fed *Oaf*-KO mice (N = 5 per group). Representative images and body weight in mice from the two groups fed a HD for 28 days (OAF injected versus saline injected) (**g**), BAT, total WAT weights and lean mass/body weight (**h**). **i-j**. Extended data from HD-fed AAV-*Oaf* overexpression model (N = 5 per group). Representative images and body weight in mice from the two groups fed a HD for total 15 weeks (**i**), BAT, total WAT weights and lean mass/body weight (**j**). Data were obtained from independent samples and analyzed using GraphPad prism 9. Data are presented as mean ± SD. (**a**), (**c**), (**g**) and (**i**) were analyzed by two-way ANOVA, all others by two-tailed unpaired Student’s t-test. ***, *P* < 0.05, “ns” indicates not significant.

## Supplementary Figure 8 OAF does not affect obesity symptoms in *Scpx*-KO and AAV-sh*Scpx* mouse models

**a**-**e**. Extended data from HD-fed *Scpx*-KO mice after OAF injection (N = 5 per group). CRISPR/Cas9 editing strategy used to generate frameshift deletions in *Scpx* by targeting exons 2 and 3 (**a**) and knockout was confirmed by Western Blot of SCPX protein (**a**). Eight-week-old male *Scpx*-WT and KO mouse were fed HD for 12 weeks; on the final day body and liver weights were determined **(c)**. Representative images and body weight in mice from the two groups HD-fed for 16 weeks (OAF injected versus saline injected) (**d**), BAT, total WAT weights and lean mass/body weight ratios (**e**). **f-g**. Extended data from an HD-fed sh*Scpx* model after OAF injection (N = 7 per group). Representative images and body weight in mice from the two HD-fed groups for 16 weeks (OAF injected versus saline injected) (**f**), BAT, total WAT weights and lean mass/body weight (**g**). **h**. Representative images of OA/PA-induced HepG2 cells after *Scpx* knockdown accompanied by OAF (200 ng) supplementation, scale bar = 200 μm. Data are presented as mean ± SD, (**d**) and (**f**) were analyzed by two-way ANOVA, all others by two-tailed unpaired Student’s t-test. “ns” indicates not significant.

## Supplementary Figure 9 Liver-specific knockdown of *Oaf* exacerbates MASLD and obesity -associated phenotypes in HD-induced C57BL/6J mice.

**a**. Eight-week-old male C57BL/6J mice were fed a HD for 12 weeks, then injected with shAAV-control (n = 5) or shAAV-*Oaf* (n = 7), and sacrificed 5 weeks after AAV administration. **b.** qPCR analysis of *Oaf* mRNA expression in different organs from two groups of mice. **c**. Representative images of livers and liver weights from two groups of mice. **d**. Hepatic TG and TCHO concentrations. **e-f.** Serum TG, TCHO, LDL-C and HDL-C of the two groups. **g.** qPCR to quantify the gene expression in liver samples from two groups. **h.** Representative images of mice at the endpoint and body weight over the 5-week period. **i.** BAT, total WAT weights and lean mass/body weight ratios at the endpoint.

Data are presented as mean ± SD. Body weight changes (**h**) were analyzed by two-way ANOVA; all other data (**b**-**g**, **i**) were analyzed by two-tailed unpaired Student’s t-test. *, *P*< 0.05, **, *P*< 0.01; “ns” indicates not significant.

## Supplementary Figure 10 Plasma OAF levels in different experimental models.

**a**. Relative circulating OAF levels in male SD rats following a single intraperitoneal injection of recombinant OAF protein (0.5 mg/kg). Blood samples were collected at 3, 6, 12, 18, and 24 h post-injection, and OAF concentrations were measured by ELISA (n=3). **b**. Serum OAF levels in male C57BL/6J mice after 8 consecutive days of daily intraperitoneal administration of saline or OAF protein (1 mg/kg/day). **c**. ELISA analysis of serum OAF levels in eight-week-old C57BL/6J mice fed a CD for 8 weeks following AAV-*Oaf* injection, (N = 6 per group). **d**. ELISA analysis of serum OAF levels in eight-week-old C57BL/6J mice fed a HD for 12 weeks, then injected with shAAV-control (N = 5) or shAAV-*Oaf* (N = 7). Data were presented as mean ± SD, **, *P* < 0.01. Statistical analysis by two-tailed unpaired Student’s t-test.

## Supplementary Table 1 The sequences of small interfering RNAs

| Genes | Sequence (5′-3′) |
| --- | --- |
| *OAF* (human) | GCUACAGCUUCGACUUCUATT |
| *SCPX* (human) | GGUAUGCAUGUGAAGCUCATT |
| *Oaf* (mouse) | GCUACACCUUUGACUUCUATT |

## Supplementary Table 2 Antibody list

| Antibodies | Source | Identifier |
| --- | --- | --- |
| OAF | Invitrogen | Cat#PA5-89072, RRID: AB_2805337 |
| MYC | Cell Signaling | Cat# 2278, RRID: AB_490778 |
|  | Proteintech | Cat#60003-2-Ig, RRID: AB_2734122 |
| SCPX | Proteintech | Cat#14397-1-AP, RRID: AB_10644332 |
| GAPDH | Abclonal | Cat# AC033, RRID: AB_2769570 |
| Ubquintin | Abclonal | A19686  RRID: AB_2862735 |
| ACC1 | Abclonal | A19627  RRID: AB_3665497 |
| ACAT2 | Abclonal | A1399 |
| SIAH1 | Abclonal | A23247 |
| IgG (Rabbit) | Abclonal | Cat# AC005, RRID: AB_2771930 |
| HRP-labeled Goat Anti-Rabbit IgG (H+L) | Beyotime | Cat# A0208, RRID: AB_2892644 |
| HRP-labeled Goat Anti-Mouse IgG (H+L) | Beyotime | Cat# A0216, RRID: AB_2860575 |
| HRP-conjugated Mouse Anti-Heavy Chain of Rabbit IgG | Proteintech | Cat# SA00001-7H  RRID:AB_2935611 |
| [Mouse Anti-Rabbit IgG Light Chain](https://rrid.site/data/record/nif-0000-07730-1/AB_2890988/resolver?q=*&l=*&filter%5b%5d=Vendor:proteintech&filter%5b%5d=Catalog%20Number:SA00001-7L&i=rrid:ab_2890988-2826444) | Proteintech | Cat# SA00001-7L  RRID:AB_2890988 |
| Coralite 488-conjugated Goat Anti-Rabbit IgG | Proteintech | Cat#SA00013-2, RRID: AB_2797132 |
| Coralite 594-conjugated Goat Anti-Mouse IgG | Proteintech | Cat#SA00013-3, RRID: AB_2797133 |

## Supplementary Table 3 The sequence of all primers in qRT-PCR assay

| Genes | Sequence (5′-3′) |
| --- | --- |
| *Oaf*-forward (mouse) | CTCTGCCCAAGGCATTAGAA |
| *Oaf*-reverse (mouse) | CGATCACGACTGTGGCAATA |
| *OAF*-forward (chicken) | CTCACATCCACAACGTCTGC |
| *OAF*-reverse (chicken) | CTGCAGCATCACGACTCTTG |
| *Hmgcr*-forward (mouse) | TCTGGCAGTCAGTGGGAACTATT |
| *Hmgcr*-reverse (mouse) | CCTCGTCCTTCGATCCAATTT |
| *Acc1*-forward (mouse) | GATGAACCATCTCCGTTGGC |
| *Acc1*-reverse (mouse) | GACCCAATTATGAATCGGGAGTG |
| *Fas*-forward (mouse) | CTGGCTGTCCCTGTCCCTAT |
| *Fas*-reverse (mouse) | TTTTGGTGGAGACGATGAGC |
| *Srebp1c*-forward (mouse) | TGACCCGGCTATTCCGTGA |
| *Srebp1c*-reverse (mouse) | CTGGGCTGAGCAATACAGTTC |
| *Srebp2*-forward (mouse) | GCAGCAACGGGACCATTCT |
| *Srebp2*-reverse (mouse) | CCCCATGACTAAGTCCTTCAACT |
| *Cyp7a1*-forward (mouse) | CTGGGGATTCGTGTAGCTGG |
| *Cyp7a1*-reverse (mouse) | AGAGCATGTCGAAACTTCCCTA |
| *Acat2*-forward (mouse) | TCATCTTCTTCGCCTTCCTGCACT |
| *Acat2*-reversed (mouse) | AGAGCCACAGCCCATCTTGATACA |
| *Fatp2-*forward (mouse) | TGTGCCAGGTCATGAGGGTCG |
| *Fatp2-*reverse (mouse) | CTCAATGGTATCTTGTATCCTCAGGAA |
| *Pparα*-forward (mouse) | AGAGCCCCATCTGTCCTCTC |
| *Pparα-*reverse (mouse) | ACTGGTAGTCTGCAAAACCAAA |
| *Scpx*-forward (mouse) | TGCCTTCAAAGTGAAAGATGGCCC |
| *Scpx*-reverse (mouse) | TTCCCTTGAAAGAAGGCCGACTGA |
| *Actb*-forward (human & mouse) | TGTTACCAACTGGGACGACA |
| *Actb*-reverse (human & mouse) | GGGGTGTTGAAGGTCTCAAA |
| *ACTB*-forward (chicken) | GATATTGCTGCGCTCGTTGT |
| *ACTB*-reverse (chicken) | TACCAACCATCACACCCTGAT |
| *SCPX*-forward (human) | TCAGCAGTGGACCAGGCATGTGTTG |
| *SCPX*-reverse (human) | TGCTTCACTGGCCAAAATTGCTGCT |

## Supplementary Table 4 Information of clinical data

# Abbreviations

| AAV | Adeno-associated virus |
| --- | --- |
| ACAT2 | cholesterol acyltransferase 2 |
| ACC1 | acetyl coenzyme A carboxylase 1 |
| acyl-CoA | acyl-coenzyme A |
| ALT/AST | alanine aminotransferase/aspartate aminotransferase |
| ANGPTL3 | angiopoietin-like 3 |
| ARSA | aryl sulfatase A |
| AUC | area under curve |
| BAT/WAT/iWAT/eWAT | brown adipocyte tissue/white adipocyte tissue/inguinal white adipocyte tissue/epididymal white adipocyte tissue |
| Baf A1 | Bafilomycin A1 |
| BMI | body mass index |
| CD/HD | chow diet/high-fat diet |
| CAMKII | Calcium-calmodulin (CaM) dependent protein kinase II |
| CNS | central nervous system |
| COVID-19 | Corona Virus Disease 2019 |
| CPZ | chlorpromazine |
| CYP7A1 | cytochrome P450 family 7 subfamily A member 1 |
| co-IP | co-immunoprecipitation |
| EE | energy expenditure |
| FABP | fatty acid-binding protein |
| FATP2 | fatty acid transport protein 2 |
| FAS | fatty acid synthase |
| FGF21 | fibroblast growth factor 21 |
| FGL1 | fibrinogen-like protein 1 |
| GEO | Gene Expression Omnibus |
| GLP-1 | glucagon-like peptide-1 |
| GPNMB | glycoprotein nonmetastatic melanoma protein B |
| GTT | glucose tolerance test |
| H&E staining | hematoxylin-eosin staining |
| HMGCR | 3-hydroxy-3-methylglutaryl coenzyme A reductase |
| ITT | insulin tolerance test |
| KO/WT | knock-out/wild type |
| LDL-C/HDL-C | low/high density lipoprotein cholesterol |
| MβCD | methyl-β-cyclodextrin |
| MANF | mesencephalic astrocyte-derived neurotrophic factor |
| MASLD/NAFLD | Metabolic Dysfunction-Associated Steatotic Liver Disease/Non-Alcoholic Fatty Liver Disease |
| MS | mass spectrum |
| MST | MicroScale Thermophoresis |
| NEFA | non-esterified fatty acids |
| OA/PA | oleic acid/palmitic acid |
| OAF | out at first |
| OD | optical density |
| PPARA | peroxisome proliferator-activated receptor alpha (in mice: PPARα; in human: PPARA) |
| qPCR | quantitative polymerase chain reaction |
| SCPX/SCP2 | sterol carrier protein X/sterol carrier protein 2 |
| SIAH1 | Siah E3 Ubiquitin Protein Ligase 1 |
| SMAD2 | Sma- And Mad-Related Protein 2/SMAD Family Member 2 |
| SPF | specific pathogen free |
| SREBP1c/SREBP2 | sterol regulatory element-binding transcription factor 1c/2 |
| T2DM | Type II diabetes mellitus |
| TCHO | total cholesterol |
| TG | triglyceride |
| THR-β | thyroid hormone receptor beta |
| TLR4 | toll-like receptor 4 |
| WB | Western blot |
| UNC5B | Unc-5 Netrin Receptor B |
